# Mixed-methods process evaluation of a residence-based SARS-CoV-2 testing participation pilot on a UK university campus during the COVID-19 pandemic

**DOI:** 10.1186/s12889-022-13792-8

**Published:** 2022-08-02

**Authors:** H. Blake, S. Carlisle, L. Fothergill, J. Hassard, A. Favier, J. Corner, J. K. Ball, C. Denning

**Affiliations:** 1 School of Health Sciences, University of Nottingham, Nottingham, UK; 2 NIHR Nottingham Biomedical Research Centre, Nottingham, UK; 3 School of Medicine, University of Nottingham, Nottingham, UK; 4 Faculty of Registrars, University of Nottingham, Nottingham, UK; 5 School of Life Sciences, University of Nottingham, Nottingham, UK; 6 Biodiscovery Institute, University of Nottingham, Nottingham, UK

**Keywords:** COVID-19, SARS-CoV-2, Universities, Process evaluation, Public health, Complex interventions, Mixed-methods, Implementation

## Abstract

**Background:**

Regular testing for Severe Acute Respiratory Syndrome Coronavirus 2 (SARS-CoV-2) is an important strategy for controlling virus outbreaks on university campuses during the COVID-19 pandemic but testing participation rates can be low. The Residence-Based Testing Participation Pilot (RB-TPP) was a novel intervention implemented at two student residences on a large UK university campus over 4 weeks. The aim of the pilot was to increase the frequency of asymptomatic SARS-CoV-2 saliva testing onsite. This process evaluation aimed to determine whether RB-TPP was implemented as planned and identify implementation barriers and facilitators.

**Methods:**

A mixed-methods process evaluation was conducted alongside the RB-TPP. Evaluation participants were students (opting in, or out of RB-TPP) and staff with a role in service provision or student support. Monitoring data were collected from the intervention delivery team and meeting records. Data were collected from students via online survey (*n* = 152) and seven focus groups (*n* = 30), and from staff via individual interviews (*n* = 13). Quantitative data were analysed descriptively and qualitative data thematically. Barriers and facilitators to implementation were mapped to the ‘Capability, Opportunity, Motivation–Behaviour’ (COM-B) behaviour change framework.

**Results:**

Four hundred sixty-four students opted to participate in RB-TPP (98% of students living onsite). RB-TPP was implemented broadly as planned but relaxed social distancing was terminated early due to concerns relating to national escalation of the COVID-19 Delta variant, albeit testing continued. Most students (97.9%) perceived the period of relaxed social distancing within residences positively. The majority engaged in asymptomatic testing (88%); 46% (52% of testers) were fully compliant with pre-determined testing frequency. Implementation was facilitated by convenience and efficiency of testing, and reduction in the negative impacts of isolation through opportunities for students to socialise. Main barriers to implementation were perceived mixed-messages about the rules, ambivalent attitudes, and lack of adherence to COVID-19 protective measures in the minority.

**Conclusions:**

This process evaluation identifies factors that help or hinder the success of university residence-based outbreak prevention and management strategies. RB-TPP led to increased rates of SARS-CoV-2 testing participation among students in university residences. Perceived normalisation of university life significantly enhanced student mental wellbeing. The complexity and challenge generated by multiple lines of communication and rapid adaptions to a changing pandemic context was evident.

**Trial registration number:**

UKAS 307727–02-01; Pre-results. ClinicalTrials.gov Identifier: NCT05045989; post-results (first posted, 16/09/21).

**Ethical approval:**

Faculty of Medicine & Health Sciences Research Ethics Committee, University of Nottingham (Ref: FMHS 96-0920)

**Supplementary Information:**

The online version contains supplementary material available at 10.1186/s12889-022-13792-8.

## Strengths and limitations of this study

**Table:** 

• This is the first study to report a process evaluation of an initiative aimed to increase student participation in SARS-CoV-2 testing in university residences. • This study provides a worked example of a pragmatic approach to process evaluation to explore the implementation of a rapid response intervention in the context of a pandemic. • A strength of the study is that the process evaluation uses both qualitative and quantitative data to illustrate intervention delivery, the facilitators and barriers to implementation and perspectives of multiple participants and stakeholders. • A limitation of the study is that the process evaluation included only a small number of students who had chosen not to take part in the scheme.	

## Background

The Coronavirus Disease 2019 (COVID-19) pandemic cause by the novel Severe Acute Respiratory Syndrome Coronavirus 2 (SARS-CoV-2) is highly contagious [1] and the world’s population is susceptible to infection [2]. If not identified and controlled quickly, an outbreak on a university campus would have potential for explosive and extensive spread, threatening the immediate and wider community. National initiatives, such as the United Kingdom (UK) Track and Trace programme [3], to target symptomatic cases and their contacts are unlikely to identify university outbreaks rapidly, as published data shows that most infections in these individuals will be asymptomatic [4, 5]. Evidence shows that individuals with minimal or no symptoms can still transmit the virus [6, 7]. Therefore, prevention of largescale virus outbreaks within the University community has required appropriate mitigation (strict personal hygiene, improved estates cleansing etc.) as well as containment (testing, contact tracing and quarantine). High-frequency surveillance testing (i.e., once or twice per week) is considered to be an effective strategy for COVID-19 disease mitigation [8, 9]. Saliva testing is one approach to both asymptomatic and symptomatic detection of the presence of replicative SARS-CoV-2 RNA, with a reported accuracy of > 99% and a sensitivity of 1–10 viral copies/μl [10]. A systematic review and meta-analysis showed that the diagnostic accuracy of saliva nasopharyngeal swab nucleic acid amplification testing (NAAT) diagnostic accuracy is similar to that of nasopharyngeal swab NAAT, especially in the ambulatory setting [11]. As such, saliva-based SARS-CoV-2 surveillance testing programmes have been operationalised in university settings in various geographical regions (e.g., [12–16].

At the University of Nottingham, asymptomatic testing has been available from an internal Asymptomatic Testing Service (ATS) since September 2020. Students arriving at the University to residences on the campus are offered asymptomatic tests (for the detection of the presence of replicative SARS-CoV-2 RNA) on arrival. A pilot study conducted during the summer of 2020 found high adherence to regular testing and acceptability of socialising via ‘household bubbles’ [16], but this was on the University’s rural campus, with the first cohort of students to occupy university residences since the outbreak of the pandemic. By Autumn 2020, the local and national situation had markedly changed [17]. Students were arriving at, or returning to, campus in a context of a second surge of COVID-19 in the UK, and the highest rates of COVID-19 in a UK higher education setting [18], with an escalating number of positive cases requiring students to self-isolate [17].

Although most students tested on arrival, the majority did not continue with regular (weekly) testing and testing uptake rates rapidly declined (participation in testing dropping from 58 to 5% [17]). This was primarily associated with fear of the negative impacts of self-isolation, loneliness and the impacts of positive test results on peers [17]. Attempts at enforcing household bubbles and other social distancing rules and regulations that had worked in a different context and environment [16] were less acceptable to students living in large traditional residences, on campuses close to the city. While approaches to the *delivery* of testing in university settings have emerged internationally (e.g., [16, 19, 20], there is limited evidence on strategies for increase testing *uptake*. At the time of writing, solutions to increasing rates of testing participation are urgently needed to inform future higher education policy and practice around outbreak prevention and management.

Clustering of positive cases has been identified in university residences (up to 31%) [20]. Recent SARS-CoV-2 transmission modelling suggests that surveillance-based informative testing strategies targeting university residences are more effective at detecting positive cases than random or voluntary testing [21]. Therefore, a novel, Residence-Based enhanced SARS-2 coronavirus Testing Participation Programme (RB-TPP) was initiated, aiming to increase the uptake and frequency of testing for SARS-CoV-2 RNA, in university residences, whilst simultaneously allowing some relaxation of social distancing restrictions within buildings.

Process evaluation is vital for understanding how interventions function in different settings, including if and why they have different outcomes or do not work at all. This is particularly important in trials of complex interventions in ‘real world’ organisational settings where causality is difficult to determine. We report a process evaluation conducted alongside the RB-TPP that explored the impact and consequences of the programme for students and staff, and established views on key aspects of the programme in order to aid better understanding of how, why and for whom such approaches and interventions are effective. This process evaluation aimed to provide insight into the value of the RB-TPP approach to prevention of COVID-19 outbreaks on university campuses. The objectives were to explore (a) the intervention as it was implemented (to ascertain the extent to which it was implemented as planned); (b) how people participated in and responded to the intervention (to ascertain the barriers and facilitators to implementation); and (c) the contextual characteristics that mediated this relationship and may influence outcomes.

## Methods

### Study design

**Fig. 1:**
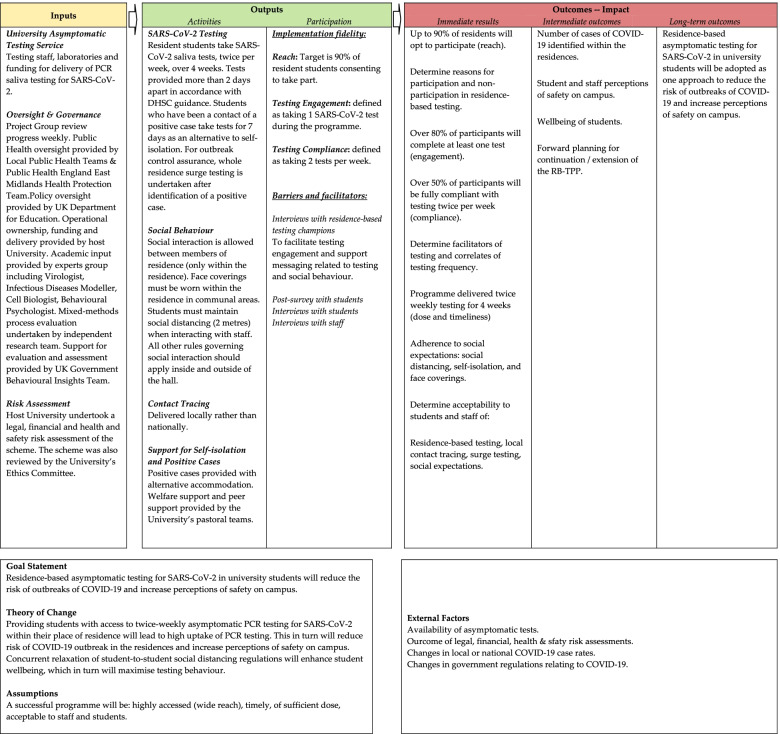
Logic Model for Residence-Based SARS-CoV-2 Testing Participation Pilot

This is a convergent parallel mixed-methods [22] process evaluation following the UK Medical Research Council guidelines [23]. Intervention fidelity is the degree to which an intervention is delivered as intended. The components of implementation fidelity evaluated here are: *Reach* (the proportion of the target group who participated in RB-TPP and their socio-demographic characteristics), *Dose* and *Timeliness* (of the intervention delivered) and *Adherence/Compliance* (of students to the minimum programme requirements). This process evaluation corresponds with the inputs, activities and outputs detailed in the RB-TPP logic model and interrogates the assumptions underlying the model and the linkages between the intervention components and outcomes (Fig. 1). The framework for documenting RB-TPP programme implementation and data sources is shown in Table 1. Terms are explained in Additional file 1. The study reporting adheres to the consolidated criteria for reporting qualitative research guidelines [24] (Additional file 2), and the TIDieR (Template for Intervention Description and Replication) Checklist (Additional file 3) [25] has been used to describe the intervention. The Checklist for Reporting Results of Internet E-Surveys (CHERRIES) [26] guided the reporting of survey findings.

**Table 1:** Framework for documenting RB-TPP programme implementation and data sources

Programme implementation questions used to guide the documentation of programme implementation	Process Evaluation Data Sources					
	Testing Service		Quantitative	Qualitative		
	RB-TPP Engagement and design	RB-TPP Implementation	Student Survey	Student Ambassador^**a**^ Interviews	Staff Interviews	Student Participant Focus Groups
**Programme design**						
1. Who were the target participants? What was the uptake and reach?	**✓**		**✓**			
2. What were the target settings? Did settings change over time?	**✓**	**✓**				
3. What theoretical model/theory-of-change were the strategies based on?	**✓**					
3. What essential elements were to be delivered in the programme?	**✓**	**✓**				
**Testing service provision**						
4. What selection process was used to identify the provider? What were the credentials of providers?	**✓**					
5. What information did the testing service providers communicate to students (what was the content and format, and were there any changes over time?)	**✓**		**✓**	**✓**	**✓**	**✓**
**Recruitment to RB-TPP**						
6. How were students recruited as participants?	**✓**					
7. What was the nature of the relationship between the student participants and the researchers or institutions involved in the programme?	**✓**					
**Tailoring, messaging, incentives**						
8. Which behavioural components are selected?	**✓**					
9. What are the reasons for that selection? (what goals are targeted)?	**✓**					
10. What, if any, other goals and strategies are proposed by programme leaders and ambassadors supporting uptake, engagement and adherence?	**✓**			**✓**		
**Programme delivery**						
11. What method was used to specify and direct the implementation?		**✓**			**✓**	
12. How long were participants involved?	**✓**	**✓**				
13. What were the testing and social behaviour expectations?	**✓**	**✓**			**✓**	
14. To what extent were the essential elements delivered? How were they monitored/measured?		**✓**	**✓**	**✓**	**✓**	**✓**
15. Were there any planned changes made to the RB-TPP while it was in progress? Why?		**✓**			**✓**	
16. Were there any unplanned changes? What happened?		**✓**		**✓**	**✓**	**✓**
**Context**						
17. What was the culture and overarching context of the participating agencies at the start of the intervention?	**✓**	**✓**		**✓**	**✓**	**✓**
18. Were there any changes/initiatives during the programme that may have affected responses to the intervention?		**✓**		**✓**	**✓**	**✓**
19. What were the immediate contextual conditions around the testing?		**✓**			**✓**	
**Participation**						
20. Who was invited to participate: numbers, locations, campuses, institution(s)?	**✓**					
21. How many potential students engaged in RB-TPP? Who were they?		**✓**	**✓**	**✓**		**✓**
22. What proportion of targeted students engaged (one PCR test) or were fully compliant (all PCR tests offered)^1^?		**✓**	**✓**	**✓**		**✓**
23. Did key people (public health leaders or topic specialists) support or advocate the programme?		**✓**			**✓**	
**Responses to programme activities**						
24. How did students participate in components of the programme?		**✓**	**✓**	**✓**	**✓**	**✓**
25. How satisfied were participants with components of RB-TPP?			**✓**	**✓**	**✓**	**✓**
26. Did students or staff identify or anticipate any changes in response to RB-TPP programme activities?			**✓**	**✓**	**✓**	**✓**
**Intervention improvements**						
27. What improvements to the intervention design and/or implementation are suggested by this data?			**✓**	**✓**	**✓**	**✓**
29. What lessons might be relevant to other interventions and settings?			**✓**	**✓**	**✓**	**✓**

*Note*: ^a^Student Ambassador Role: peer-to-peer recruitment (engagement) and implementation of social distancing (adherence). Testing: ^1^non-invasive saliva polymerase chain reaction (RT-qPCR) tests for SARS-CoV-2 RNA with samples collected and analysed in the University’s laboratories

### Study setting and participants

A total of 588 registered university students were listed occupants in two, similar, mixed-gender residences on a single UK university campus at the start of the study (April 2021) and eligible to participate (site 1: 366, site 2: 222). Of these, 116 were not living onsite (by choice) due to the COVID-19 pandemic (e.g., they had not physically returned to the university campus during the pandemic) and therefore did not take part in the RB-TPP. There were 472 students living onsite at the time of the study (80% occupancy; site 1: 311, site 2: 161), of whom 464 provided written informed consent online, to take part (98%; site 1: 306, site 2: 158). There was a two-step approval process to take part in RB-TPP. First step was an online privacy notice, which contained consenting to have saliva samples tested for presence of SARS-CoV-2 RNA and, if positive, for follow-up analysis of that sample to include virus sequencing but excluding any human DNA sequence analysis. This also asked if participants would be willing to be approached for the purposes of research. Next, there was a separate online consent form for research participation. Students who opted out of the programme were re-located to alternative temporary accommodation during the study period. Reasons for decline were perceived risk for COVID-19, and inconvenient timing of the programme due to its proximity to academic examinations. The settings were deemed to be more ‘traditional’ residences with large corridors, shared facilities, communal dining and socialising models and where a prior COVID-19 mitigation approach of the small group ‘student household’ (e.g., in [16]) was less relevant. Eligible process evaluation participants were in-house students who had either taken part in or opted out of the RB-TPP, and staff with a role in intervention delivery or student support.

### The intervention: residence-based asymptomatic testing participation pilot (RB-TPP)

The aim of the RB-TPP was to increase and maintain participation of students in regular testing for SARS-CoV-2 RNA, in university residences. The RB-TPP (Fig. 1) was planned for delivery over 4 weeks in April–May 2021 and required asymptomatic students to take a saliva test to detect SARS-CoV-2 RNA, twice weekly for 4 weeks. This was combined with relaxed social restrictions within the residence during the study period (i.e., removing the need for 2-m distancing between students living in the same residence), devolved local contact tracing (i.e., contacts traced locally by a university and local public health team, rather than the national Track and Trace service) and enhanced support for students who were required to self-isolate. The processes were agreed with NHS public health partners and were in alignment with UK law. All students had access to usual university welfare support systems (e.g., welfare / disability advisors, a university Student Hardship Fund (standard provision) and Student Crisis Fund (additional provision during the COVID-19 pandemic) for students experiencing financial difficulties, counselling services, etc). Specific additional support for self-isolating students included attendance to food and medication needs, provision of telephone support for students, and telephone reassurance for close relatives (if requested by the student, to ensure confidentiality was observed). A dedicated email helpline was established for enquiries, with ATS service staff providing responses Mon-Fri 09:00–17:00. Any identified concerns relating to student welfare were raised with student welfare teams and/or academic tutors as appropriate. Students were required to wear face coverings when interacting with staff (e.g., in dining rooms), although face coverings were optional in areas of the residence where students were mixing only with other students (e.g., in student bedrooms or social areas). The identification of any individuals testing positive for SARS-CoV-2 RNA during this period would trigger residence surge testing, whereby all students living in the hall would then be required to test daily for 7 days. Additional surge testing was available for contacts of positive cases outside of the residences, if required (e.g., for academic cohorts if the student had been attending face-to-face teaching sessions).

Communications with students were focused on expectations (testing and social behaviour), testing processes and logistics. Communications were delivered primarily by email, supported by face-to-face communications from staff with student-facing roles, and three student ambassadors known as ‘testing champions’ offering peer-to-peer support and encouragement to participate. The testing service provider was the host University’s flagship asymptomatic testing service (ATS), which at the time of writing was one of only eight laboratories specifically recommended for, or accredited for, SARS-CoV-2, in the UK. The views of university staff and students towards the ATS have been published elsewhere [16, 17]. Tests were non-invasive self-administered saliva tests that were collected and analysed in the University’s laboratories. Specifically, they were reverse transcription polymerase chain reaction (RT-qPCR) tests that included the assay controls for the qualitative detection of viral RNA from SARS-CoV-2 in saliva specimens (since SARS-CoV-2 is an RNA virus, the genetic material for SARS-CoV-2 is encoded in ribonucleic acid (RNA)). During the study period, a second confirmatory National Health Service (NHS) Pillar 2 test was required for all SARS-CoV-2 RNA positive individuals identified via asymptomatic tests (no longer required from July 2021). The testing service set up deployments in the dining areas of the two sites, which were staffed on Tuesdays and Fridays for the students to drop off their samples. Opening hours were initially 10:00–14:00, but hours were extended to give students greater flexibility in when they could drop off their samples – extending hours initially to 16:00, then to 19:00 after student feedback. All RB-TPP processes were overseen by the university COVID-19 Testing Operations group working in collaboration with local and national public health teams.

### Data collection

**Fig. 2:**
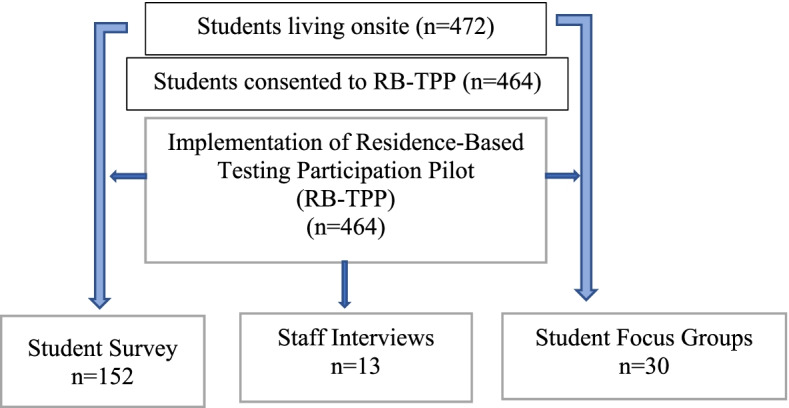
Process Evaluation Data Collection

We gathered quantitative measures of intervention activities (such as number of students participating in the residence-based testing participation scheme) [27], and qualitative exploration of the interaction between the programme, how students and staff experience it, and the contextual characteristics of the two sites in which it was delivered. This is detailed in Table 1 and Fig. 2.

### Implementation fidelity

#### Reach

Program reach was evaluated through the number of students recruited into the RB-TPP compared to the number of potentially eligible students living in one of the two participating sites in April 2021 (identified via residence manager’s records). Recruitment data were collected by the COVID-19 Testing Operations Team and entered directly into a secure web-based database. Reasons for participating (or not), and characteristics of students opting in and out were collected in an end-of-programme survey.

#### Adherence/compliance

Participant compliance was defined as the proportion of students that completed two tests per week. Pre-defined compliance was therefore completion of 8 tests over the 4-week period. Objective data on uptake were recorded by the COVID-19 Testing Operations Team and student self-reports of reasons for compliance and non-compliance with testing, and adherence and non-adherence to pre-defined behavioural expectations were collected in an end-of-programme survey.

#### Dose and timeliness of intervention delivery

Data related to the dose delivered (number of tests offered, total duration of intervention) and timing of intervention delivery (when tests were available, when and how results were received, additional surge testing, contact testing). Data were recorded by the COVID-19 Testing Operations Team with any reasons and challenges raised explored in the end-of-programme survey.

### Online survey

Students opting in or out of the intervention received an email at programme end, sent from the ATS, containing a direct link to an information sheet and online survey (Additional file 4), hosted on Jisc Online Survey platform with automatic capture of responses. Items were adapted from a prior study [16] and contained a mixture of closed and open-ended free-text questions. The survey contained approximately 2–4 items per page, over 19 pages. Adaptive questioning (certain items, or only conditionally displayed based on responses to other items) was used to reduce number and complexity of questions. Non-response options were included, participants could amend their answers up to the final page, and completeness checks occurred prior to submission using JAVAScript. Usability and technical functionality of the electronic questionnaire were tested before fielding. Those who volunteered to take part in the survey received a £5 gift voucher as compensation for their time. The survey contained 55 items in 4 sections. Section 1 included 16 categorical items relating to socio-demographics (age, gender, ethnicity, international/home student, work status, stage of study, accommodation type), and prior experiences of COVID-19 (self/others). Section 2 included 25 items relating to confirmation of participation (or not) in the RB-TPP, reasons for taking part, number of tests taken (uptake/engagement) and adherence (testing according to protocol), views towards the intervention (testing and follow-up, logistics, communication, plans for surge testing, adherence to social behaviour regulations during the intervention), and experiences of self-isolation and support. These items were a mix of categorical and open-ended questions. Section 3 included 7 items relating to COVID-19 risk perception before and after the intervention (rated on a scale of 1 = ‘I didn’t think I would get it’ to 10 = ‘I knew I would most certainly get it’), views towards COVID-19 protective behaviours (social distancing, face coverings, handwashing, self-isolation - rated from 1 = not at all important, to 10 = extremely important). Worry about self or others contracting COVID-19 over the past 2-weeks were assessed on 4-point scales (‘do not worry...’, ‘occasionally worry...’, ‘spend much of my time worrying...’, ‘spend most of my time worrying...’). Anxiety was measured by the Generalised Anxiety Disorders Scale (GAD-7 [28, 29];. The GAD-7 is commonly used 7-item measure of general anxiety symptoms across various settings and populations, with established reliability and validity in clinical and non-clinical populations [28, 30], and university student samples [31–33]. The scale has shown high internal consistency (Cronbach’s alpha 0.83) [33]. The GAD-7 total score is calculated by assigning scores of 0, 1, 2, and 3, to the response categories of ‘not at all’, ‘several days’, ‘more than half the days’, and ‘nearly every day’, respectively, and summing item scores. Scores of 5, 10, and 15 are taken as the cut-off points for mild, moderate, and severe anxiety, respectively. Using the threshold score of 10, the GAD-7 has a sensitivity of 89% and a specificity of 82% [29].

### Student focus groups and staff interviews

The qualitative arm of the study aimed to explore the impact, consequences and experiences of the staff and students involved in the RB-TPP and establish their views on all aspects of the programme and the implementation fidelity components detailed above. Students’ perspectives were examined through seven focus groups planned to take place at the end of week 2 (mid-point, May 2021). This included six groups for students (opting in or out of the RB-TPP), and a single group for student ambassadors (‘testing champions’). Group size ranged from 3 to 6 attendees. All students received £20 as compensation for attendance at a focus group. Staff perspectives were examined through individual interviews planned to take place in week 4–5 (end point, June 2021). Eligible participants were purposively sampled according to role (testing operations, residence management, student support). They were contacted by email, provided with a study information sheet and invited to take part in a focus group or interview. Student focus groups were conducted by two researchers (SC, LF) and lasted between 42 and 61 minutes (mean 52 minutes). Staff interviews were conducted by one researcher (LF) and lasted between 15 and 35 minutes (mean 22 minutes). Researchers collecting and analysing data had no involvement with the testing service or university residences. All participants in focus groups or interviews received an information sheet and provided written informed consent online, prior to the interview (additional to consent provided for participation in the RB-TPP). Interviewers used question guides (Additional file 5) developed by the lead author, a health psychologist, in consultation with the process evaluation team and members of a patient and public involvement and engagement (PPIE) group, and field notes were taken. The guide included prompts to discuss opinions about programme content, dose, delivery style, and delivery mode, as well as perceived benefits of and barriers and facilitators to participation. All interviews and focus groups were held via a video-conferencing platform, audio-recorded and transcribed.

### Barriers, facilitators and acceptability

Barriers and facilitators to implementing RB-TPP, and acceptability of the programme to students and staff were identified through the student focus groups and survey, and staff interviews as detailed above.

### Participant and public involvement

Student and staff views informed the study design and interview questioning guides at the point of study conception, via a Participant and Public Involvement and Engagement (PPIE) group. Students expressed a preference for small (*n* < =6) focus groups, and staff preferred to participate in individual interviews. Study findings will be disseminated to all participants through this publication and lay summaries disseminated via the participating university.

### Data analysis

Quantitative survey data were analysed using descriptive analysis and non-parametric tests of association (Spearman’s Rho, Kendall’s Tau, and Chi-Square). Qualitative data from the semi-structured interviews and focus groups were analysed by two researchers (SC, LF) using deductive and inductive coding [34]. First, coding was guided by the assumptions of the RB-TPP programme logic and key components of the intervention. This included: impact of the RB-TPP on containing the spread of COVID-19, impact on students (e.g., personal risk and wellbeing, satisfaction, social behaviours, testing processes, identifying cases, wellbeing etc), impacts on staff (e.g., personal risk and wellbeing, satisfaction, resources etc), views towards key components of the pilot (e.g., communications, ambassador role, testing process, test type, logistics, social aspects, surge testing, enhanced contact tracing, managing positive cases, isolation support), barriers and facilitators to implementation and outcome (e.g., social factors, government policy, new guidance, incidents) and future recommendations. Then an inductive approach was used to code relevant features of the data beyond the pre-defined categories. Coding was undertaken using NVivo 12 software (released March 2020) [35]. Discrepancies were discussed until consensus was reached. Themes were identified from the codes and mapped to the Capability Opportunity Motivation – Behaviour (COM-B) model [36]. This model categorises behaviour (B) as the result of an individual’s capability (C); opportunity (O); and motivation (M), to perform the behaviour. The behaviours of interest for this evaluation were: (i) participation in the RB-TPP programme (student participants); and (ii) delivery of the RB-TPP (staff participants).

## Results

Data were all collected from programme end. Student focus groups were conducted within 1 week, and staff interviews within 3 weeks. The survey closed after 3 weeks. Monitoring data relating to uptake and reach were collected concurrently. Due to a national escalation of positive cases of the COVID-19 Delta variant at the time, the primary behavioural element of the RB-TPP (relaxed social distancing rules) was terminated (after 10 days, broadly the mid-point). Due to a need to focus on the institutional response to the changing national picture, testing compliance data for Site 1 and Site 2 were therefore collected only from Thursday 6th May to Sunday 16th May 2021, although the testing provision was retained. Therefore, for these purposes, any student who had tested three times or more over this period was therefore classed as fully compliant.

In this process evaluation, 152 students completed the online survey (88 women, 63 men, mean age 19.24 years; SD = 1.34) of whom 145 (95.4% of survey respondents, 31% of RB-TPP participants) had participated in the RB-TPP. All responses were included in analysis. Total survey participation rate was 34% of RB-TPP participants. Survey participants were broadly representative of RB-TPP participants. There was a total of 30 students (14 women, 16 men, mean age 19.9 years; SD = 2.31) attending one of 7 focus groups, and 13 staff (7 women, 6 men) were interviewed. Staff job roles were related to the testing service (strategic and operations), hospitality, accommodation or other student support. The staff interviewed were student facing (e.g., welfare support, hall managers, domestic staff with direct student contact) (*n* = 6) and non-student facing (e.g., service operations staff without direct student contact) (*n* = 7).

### Implementation fidelity

#### Reach

A total of 464 students chose to participate in RB-TPP (site 1: 306; site 2: 158). This represented 98% of students who were resident in the two sites at initiation of the RB-TPP (and 79% of all students listed as occupants, including those who were not present on campus at the time of the study). Survey participant characteristics are presented in Table 2, reasons for participation are shown in Table 3. There was no significant difference in the proportion of participants consenting to the RB-TPP at site 1 and site 2, or in sociodemographic characteristics of those who opted in, or out. Further details of student health and prior experience of COVID-19 are provided in Additional file 6.

**Table 2:** Characteristics of RB-TPP participants

	n(%)		
	Total sample	P^a^	Non-P^b^
**Gender**	*N* = 151	*n* = 144	*n* = 7
Female	88 (58.3)	86 (59.7)	2 (28.6)
Male	63 (41.7)	58(40.3)	5 (71.4)
**Ethnicity**	*N* = 150	*n* = 143	*n* = 7
White	129 (86.0)	123 (86.0)	6 (85.7)
Mixed	5 (3.3)	5 (3.5)	1 (14.3)
Asian or Asian British	11 (1.3)	10 (7.0)	
Black or Black British	2 (1.3)	2 (.7)	
Middle Eastern or Middle Eastern British	1 (.7)	1 (.7)	
Prefer not to say	2 (1.3)	2 (1.4)	
**International Student**	*N* = 151	*n* = 144	*n* = 7
Yes, European Union	9(6.0)	9 (6.3)	0
Yes, International	6 (4.0)	6 (4.2)	0
No, Home Student	136 (90.1)	129 (89.6)	7 (100)
**Year of Study**	*N* = 151	*n* = 144	*n* = 7
Foundation	2 (1.3)	2 (1.4)	0
1st	140 (92.7)	135 (93.8)	5 (71.4)
2nd	2 (1.3)	2 (1.4)	0
3rd	1 (.7)	0	1 (85.7)
4th	3(2.0)	3 (2.1)	0
5th	0	0	0
Postgraduate	3 (2.0)	2 (1.4)	0
**Accommodation during term-time**	*N* = 151	*n* = 144	*n* = 7
University Halls of Residence	150 (99.3)	144 (100)	6 (85.7)
Temporary, alternative accommodation	1 (.7)	0	1 (14.3)

*RB-TPP* Residence-Based Testing Participation Pilot

^a^PIP: Participated in RB-TPP (opted in)

^b^Non-P: Did not participate in RB-TPP (opted out)

**Table 3:** Reasons for participation in the RB-TPP (n = 145)

What were your main reasons for taking part?	% (n)
To contribute to national efforts to contain COVID-19	58.6 (85)
Helping to keep campus safe for everyone	56.6 (82)
Getting to know other students better	51.0 (74)
Being involved in COVID-19 research	39.3 (57)
To protect myself	36.6 (53)
To reassure myself about my health status	35.2 (51)
To protect local communities	29.7 (43)
To reassure my family about my health status	23.4 (34)
To protect my family	22.8 (33)
Having pride in my university	17.2 (25)
Getting to know staff better	7.6 (11)
Learning something new about COVID-19 itself	6.2 (9)
Learning something new about COVID-19 testing^†^	6.2 (9)
Other	5.5 (8)

% of respondents who selected each answer option out of those who indicated they participated in the RB-TPP ^†^ Saliva-based test for the detection of SARS-CoV-2 RNA

Those that specified ‘other’ took part for the following reasons: to be able to socialise with other students; for reasons of practical convenience (to remain in the RB-TPP residences); to protect academic staff during teaching sessions

Of survey respondents, 24 students undertaking paid or voluntary work, of whom 12 identified themselves as key workers (e.g., health or social care, food chain supplies, public service), regularly coming into close contact (< 2 m) with others outside of the residence. Although reasons for participation were diverse, the three most common reasons for participating were positive: to contribute to the national efforts to contain COVID-19, helping to keep campus safe for everyone, and getting to know other students better (Table 3). A minority participated to avoid negative consequences (e.g., perceived pressure, not wishing to relocate during the intervention). Seven survey respondents had opted out of the programme (5 M, 2F;1 keyworker). Over one third of survey respondents had tested positive for COVID-19 at some point during the pandemic and 83.2% had been required to self-isolate at least once before. Of those who opted out, only two had previous experience of self-isolating and none had tested positive for COVID-19 previously. All 7 students reporting an existing physical health issue had participated in the pilot. One fifth of the sample (*n* = 29, 19.3%) reported a prior history of mental health issues. All the survey respondents reported symptoms of anxiety on GAD-7, and this was moderate to severe in 45.9% (*n* = 68) students who met the screening threshold for general anxiety disorders (score > =10 on the GAD-7 [28, 29];).

### Adherence/compliance

#### Saliva testing

Intervention monitoring data showed that 409 of the 464 intervention participants (88%; site 1: 278, site 2: 131) completed at least one test during the data collection period. A total of 213 (site 1: 134, site 2: 79; 46% of all participants; 52% of those who tested) were classed as fully compliant. There were no reactive tests identified. Of the 145 respondents, 64.1% (*n* = 93) were extremely and 33.8% (*n* = 49) somewhat confident in the results of their asymptomatic saliva tests. Almost all students were satisfied with the physical process of taking a saliva test (95.9%, *n* = 139/145). Non-compliance was largely due to students being away, or missing drop-off times due to academic commitments (prior to the timings being extended to address the raised issue).

#### Correlates of testing frequency

A larger number of tests completed was associated with increased satisfaction with their ability to interact with others in their hall (*r* = −.180, *p* = .031, *n* = 145), although the magnitude of this association is small. Students who were more satisfied with the test drop-off and pick-up processes were more likely than those dissatisfied to report full test compliance during the pilot (*X*^*2*^ *(1, n = 143)* = 4.917, *p* = .027, effect size (Cramer’s *V*) = .185). Students who reported higher levels of worry about the risk of getting COVID-19 completed a greater number of tests during the pilot (ie., were more adherent to testing) (*r* = −.151, *p* = .043, *n* = 138). A higher number of tests completed during the pilot was significantly associated with increased positive perceptions towards social distancing (r = .178, *p* = .033, *n* = 144), face coverings (r = .227, *p* = .006, *n* = 144), and hand washing (*r* = .165, *p* = .047, *n* = 144) as essential controlling measures for COVID-19.

#### Social behaviours: social distancing, self-isolation, and face coverings

Of respondents, 88.3% (*n* = 128/145) were somewhat or extremely satisfied with level they were able to interact with other people in their hall of residence during this time. Almost all (97.9%, *n* = 141) felt that relaxed social distancing in halls was acceptable; many indicated that social contact was happening regardless and better to be sanctioned: ‘*we already have unavoidable contact’*. Some indicated that the relaxation of social distancing and being able to socialise more freely was a reason for their participation. There was an overwhelming perception that the benefits of social interaction to mental health outweighed the risk of virus transmission which was perceived to be low. Three-quarters (75.5%, *n* = 108) of students reported always maintaining social distancing (two metres) when interacting with staff, out of ‘*courtesy’* and *‘respect’*. An estimated 92.4% (*n* = 133) indicated that they had adhered to social distancing and all other COVID-19 security rules outside the hall environment ‘*to protect others*’. All (100%) students reported they did not have to self-isolate during the pilot (*n* = 144). Three-quarters (73.4%, *n* = 80) of students indicated they were either ‘extremely’ or ‘somewhat satisfied’ with the University’s support offer for students required to self-isolate. Over half (7.3%, *n* = 82) reported always wearing a face covering in communal areas in their hall. Non-compliance with behavioural regulations (e.g., face coverings, social distancing) was largely due to misunderstanding (of students and some staff) as to what was allowed and where, and frequent changes or inconsistency in messaging. Complacent attitudes and misbehaviour of some students caused frustration among those conforming to the rules.

### Student satisfaction, barriers and facilitators

Of respondents, 88.1% (*n* = 126) would take part in a similar surveillance testing participation scheme in future. Eight out of ten respondents (82.5%, *n* = 118) would recommend it to their peers. Dissatisfaction in the minority largely stemmed from communication issues (with regards conflicting information, perceived pressure or hostility), and a mismatch between some students’ expectations of the pilot and the reality of delivery (with regards incentives, and social behaviour regulations). Barriers and facilitators to participation in, and delivery of, RB-TPP are mapped to the COM-B framework [36] (Table 4).

**Table 4:** Barriers and facilitators to participation in and delivery of RB-TPP, mapped to the COM-B Framework

COM-B	Facilitator or barrier to participation and delivery	Student perceptions: participation in RB-TPP	Theme	Staff perceptions: delivery of RB-TPP
***Capability*: physical and psychological capacity to engage in the behaviour**	Facilitator (physical)	“The saliva test was really, it’s really easy to do and it’s not like uncomfortable like the swab tests so, yeah, I much prefer doing them.” [FG2, S4] “It just seemed really well organised, easy to find and easy to do. “[FG1, S5]	Test ease and preferences	“I think making it easy for them reduces the barriers and makes it more likely that they’ll get involved with us.” [I, S4] “We like to say that our testing protocol is a lot easier and less invasive than the one you stick up your nose, it takes longer to get the results, but they are more accurate. But that’s not seen a good enough reason to test” [I, S1]
	Facilitator (psychological)	“Because that’s [socialising] the whole point of coming to university” [S “I would just say it felt a little more normal, like there was one bubble, our hall within uni that we could live almost as if Covid wasn’t really a thing, obviously there was the testing, mask wearing in communal areas and the dining hall, but other than that it was as [S1] said like, as close to university experience as we could get.” [FG1, S4] “I’ve really enjoyed being in Halls, obviously meeting people, making friends and in general the Hall living experience has been as I hope it would be normally” [FG4, S1] “It gave a sense of normality because being in university halls and not allowed to socialise is not a good experience.” [S]	Perceived normality of university life	“I think it was the pilot, they did see the kind of freedom of it, so I would guess their kind of normal student experience kind of came back into play a little bit and they did see that kind of happen.” [I, S7]
	Facilitator (psychological)	“I think it was a big positive impact on wellbeing. I know a few people that were really anxious about socialising anyway before the pilot and they were much more comfortable while it was happening. They seemed to be much more relaxed and happier in themselves.” *[FG7, S1]* “I mean other than being happier, it was a relief that we could actually communicate with other people...I was very happy that you know, it occurred.” [FG1, S1] “I went to dinner alone for 5 months straight, until this pilot study came in. It was definitely less isolating”. [S] “Felt nice for normality and to speak to people I’d seen around but never been able to speak to, definitely improved my mental health” [S] “more is risked by allowing students to feel isolated in their tiny rooms with only 5 in their household and letting their mental health plummet” [S]	Student wellbeing	“I haven’t spoken to students about it but I think that it would have helped their mental welfare in terms of being able to socialise more freely” [I, S12) “It didn’t really make a difference, I don’t think, because they’ve been partying all year so, you know, there have been quite a few incidents round the campus but they have been partying all year. So I don’t think it made that much of a difference to them.” [I, S9]
	Facilitator (psychological)	“[before the pilot] They didn’t really want to go out of their way to get tested, especially as they’d been through the really severe isolation, they weren’t allowed to leave at all, and they don’t kind of want to go through that again.” [FG1, S5] “[before the pilot] I think it was a very real fear of it’s just not going to be a good time if you quarantine” [FG4, S2] “[with surge testing in place]...they don’t want to be the reason that the rest of their household has to stay indoors for 10 days and not see anyone or do the things that they’ve got planned. So I think the surge testing was actually quite a good idea to kind of test to see how the spread would happen.” [FG7, S2]	Reducing fear of self-isolation	“I suspect beforehand there would have been a bit of taboo almost about going and testing at the risk of them becoming positive and then locking down households. If you think in a house of six, one of you goes, gets tested and then the rest of them have to isolate, the other five might be a little bit miffed at that.” [I, S4] “And then we’ve got the process in place now, if we do have a positive we don’t have to isolate the whole household, you know, that person then just gets moved to the [alternative accommodation], taken out the equation, and then everybody just has to test for one week and then negatives are coming back in 24 hours, so really they don’t have to isolate for very long, it’s only a 24 hour period where at the beginning of the academic year we had 97 and 92 households in isolation for ten days.” [I, S3]
	Barrier (psychological)	“They [other students] said, ‘well I don’t see the point in testing because there’s no cases on campus anyway so why should we test?’” [FG3, S1] “It felt basically the same from my point of view. I think other people did mix more but, yeah, they hadn’t really been enforcing the household only mixing beforehand anyway, everyone had just been sitting in the dining room next to each other for a while before, so it didn’t feel like it changed that much” [FG2, S4] “It needs to be a bit more forceful in ‘look, you’re either doing this or you’re not’, because otherwise I think that kind of – what’s the word – just that indifference will set in and people stop doing it.” [FG2, S5]	Complacency and indifference	“...there was some feedback that some students really didn’t quite get the point of why we were doing it, why they were doing the testing” [I, S12] “The students weren’t aware of the bigger picture here what was going on really, because they’re obviously in their own household, they’re on campus, they’re in their little bubble and really it’s a much bigger thing that we’re trying to protect.” [I, S3]
***Opportunity*: external factors that make the behaviour possible**	Facilitator (testing service)	“now since it’s [test site] literally just inside the Hall, so it’s a lot more convenient.” [FG3, S6] “It just seemed really well organised, easy to find and easy to do. “[FG1, S5] “test results were always given out quickly” [S]	Convenience and efficiency of testing	“The very fact that we were in the Halls of Residence in a location where the footfall was really good, people were going past all of the time, I was surprised how important that was and make it easier for people to drop samples off, yeah, I was surprised about that.” [I, S2] “Well I think the testing team are brilliant. So I think anything relating to the process of the testing was always done brilliantly and they would adapt to whatever”. [I, S12] “the testing team were really good on site as well, you know, they’d just turn up and say ‘we’re here for the kit’ and off they go, they were really good, showed us how to use all the laptops, how to record the samples.” [I, S3]
	Facilitator (testing service)	“I also appreciated how transparent the university was with testing figures” [S] “It was pretty clear and we got regular updates on the progress of the participation” [S]	Transparency	“.. keeping the comms a bit more bitesized and even if there had have been, if we’d had the time to do infographics and things” [I, S7]
	Facilitator (testing service)	“I thought it was pretty straight forward actually, the information everything, um … the emails were pretty frequent everything like that, so I think we had all the information that we needed.” *[FG1, S1]* “I definitely want to know what’ll happen if someone tested positive in the Hall, what that means for me.” [FG2, S5] “I feel everything was well communicated, if any changes occurred, we would be notified about these very quickly, we would always be reminded to collect and hand in our test samples. So overall, information was very well communicated”. [S]	Clear and regular information	“I think the university’s messages are really good, it’s just that they’re having to respond to changes that have come from government.” [I, S11] “The communication team did well communicating what they had to communicate but for ever changing times, needing to clarify behaviours and everything just led to excess communications being sent out and I imagine that didn’t go down too well with students.” [I, S6]
	Facilitator (peers)	“It seemed as far as I was aware, as I was aware, everyone who was in halls was testing” [FG1, S4] “during the pilot the adherence was pretty good … I think overall we did pretty well with everyone testing and everything like that.” [FG1, S1]	Testing as a social norm	“...going into the new academic year when you’ve got a new Halls intake, pushing normalisation of testing I think is really beneficial potentially.” [I, S4] “The positive of the pilot was that we had far greater, a far greater amount of testing within them two halls than we’ve seen across accommodation at any point. I think we got 60, 70% within the halls which just wouldn’t have happened without the pilot so that was the positive.” [I, S6]
	Facilitator (peers)	“it was a great opportunity to be able to actually meet other people in, um, in a more secure environment and without having to breach Covid restrictions, um or kind of standing outside in the rain talking to people” [FG1, S5] “I’ve really enjoyed being in Halls, obviously meeting people, making friends and in general the Hall living experience has been as I hope it would be normally” [FG4, S1]	Creating social opportunities	“There was positive impact because we definitely seen them socialising more, we opened up more of our areas so they could socialise, we opened the bar up at [site 2] for use so they could use the bar as a bar service throughout the period, so they gained more facilities for starters, more socialising facilities and they can socialise with more people.” [I, S3]
	Barrier (testing service)	“... the submitting part is the hardest part because the timing isn’t really suitable for people who’ve got live sessions” [FG3, S6] “I saw a lot of people handing in tests but then not being given another one to do” [FG7, S1] “People would go home without telling the uni and they would be counted as not having a test despite them not being able to” [FG1, S3]	Logistical challenges	“we learned as we went through the timings that we were there – with the greatest of respect there wasn’t a lot of point in us being there at 10 am in the morning! There was a lot of benefit from us being there at five o’clock in the evening when it was the first opening of the doors for dinner. And that was an ideal time. And we learned that and perhaps we should have thought of that right at the very start.” [I, S2]
	Barrier (testing service)	“information about the timing and place for picking up and dropping off tests kept changing” (S) “I didn’t feel like it was very clear, like, the extra things we were allowed to do, we were just sort of, it felt like we were told, like, vaguely what we were allowed to do but it wasn’t very clear” [FG2, S4] “Personally in [site 1] we had a couple of incidents where testing times weren’t clearly communicated between the testing team and the students.” [FG1, S5] “The second day of pilot we were in a room with eight people who were all in [site 1] and we got kicked out by a security guard. When I asked him why because we were doing a pilot scheme he said ‘I don’t know, I’ve not been told about it’.” [FG5, S2] “Felt like there was a lot of miscommunication between security and the organisers as security kept trying to enforce normal social distancing rules”. [S]	Changing processes and mixed messages	“If you’re changing the goalposts every week, that gives my team a really tough job because you’re having to make people understand something new every week and they’re just going to eventually switch off because you’ve only got a few opportunities.” [I, S6] “I think if it was to be in halls again, it’s just making things much more black-and-white so that the students know exactly what their rules are and what the expectations are and not setting the expectation crazy high.” [I, S7] “You need to have a consistent message throughout for that to get through to people and for people to really understand it” [I, S6]. “There was a bit of confusion over what they were allowed to do, what they weren’t allowed to do, and you’ve got two security people, you’ve got university security, you’ve got [company name] and I’m not sure they were always on the same page and I’m not sure security was comfortable with going in and dealing with stuff.” [I, S4]
***Motivation*: brain processes that direct behaviour, such as decision-making, habitual processes and emotional responses**	Facilitator (peers)	“The reason why I wanted to do it was to be able to mix with more people in my Hall, get to know people better, so yeah, it was largely sociable reasons” [FG2, S1] “I wanted to be able to socialise more freely within Halls like with friends and stuff” [FG4, S4]	Social motivation	“So it’s about telling them why they should be tested every day so they can socialise and have a more normal university life.” [I, S9]
	Facilitator (peers)	“That was probably the main benefit of it was just, you know, just easily being able to text and answer a question.” [FG7, S3] “I think it definitely had its place as a tool to remind people” [FG7, S1]	Peer-to-peer support (student ambassadors)	“We had champions recruited to help and I think they were great because ... it’s very important to know what’s the view on the ground from their perspective and having those testing champions was a fantastic link to be able to understand what was going on from the students’ point of view.” [I, S8] “it’s showing that the staff and students are working together and it shows student voice and also where the authority or the boundaries are. Involving people, I think is the key.” [I, S11]
	Facilitator (staff)	[punitive] “My reason for taking part is because I had a guy, there was just some guy that was coming round knocking on everyone’s doors saying that we’d be relocated from the Hall if we didn’t participate, so that sort of scared me into doing it.” [FG4, S4] [punitive] “communications about the Pilot were partisan; there was immense pressure to participate”. [S]	Practical motivation	Anything from staff to suggest they thought students felt under pressure to take part, or weren’t? Staff didn’t mention the feeling of being under pressure to participate
	Facilitator (self)	“I really felt the relaxed restrictions during the testing pilot helped um, where mixing between households was inevitable, I felt it was probably a lot safer during the pilot” [FG1, S2] “I felt safe knowing that everyone had been tested.” [FG6, S2] “Felt safe interacting knowing there were no cases in the hall” [S] “I feel safest on campus than anywhere else” [S] “it did feel a bit unsafe as it’s quite a big hall and knowing anyone could mingle and get ill was concerning” [S]	Safety perception	“Yeah, I think it’s a good idea (testing). Like I say, it made the staff feel safe, it made us feel safe, and I’m hoping it made the students feel safe as well. There are a lot of students who are sensible and behaved, so I’m hoping that it made them feel safe as well.” [Staff member
	Facilitator (community)	“... by testing twice a week, if then I’m then at less risk as well if I’m negative of passing it on to other people, so yeah, that was it for me.” [FG2, S1] “And then with the testing twice a week I suppose if there was a case it would be caught more quickly and then if there was an outbreak it could be contained quicker as well.” [FG2, S2]	Societal responsibility	“I think it’s very important to controlling the virus within universities. It keeps us ahead of the virus. In terms of knowing when an outbreak has occurred, it gives us more time to get it under control so I think it’s an important part of getting universities back to you know some kind of normal.” [I, S6]
	Facilitator (university)	“quite liked the incentives they gave us, like, with the stats and also the little goody bags they gave us, that was pretty good.” [FG4, S5] “It was only until it was known that there was food bags when students started to get tested.” [S]	Incentivisation	“At the end of the day I think it’s wrong to incentivise it, because I think that people should be doing it as a norm, and I don’t see a problem in doing it, as normality is once a week you do a test. To me that doesn’t seem like we’re asking anything really hard of anyone to do, but it’s a shame that we have to incentivise things in that way.” [I, S10]
	Barrier (university)	“the food bags weren’t enough. It wasn’t like enough but it didn’t encourage them to get out of bed to drop off their samples and stuff.” [FG6, S2]	Lack of incentivisation	“And the incentive wasn’t there for them because they’d already been mixing all year, you know, we’re always fighting the fight, you know, ‘no you’ve got to split up, no you’ve got to stay together in your household’, so from the start it’s been difficult for us that way and there was no massive incentive for them” [I, S3] “I don’t think the students felt that it [food bag incentive] was impactful enough for them. They didn’t feel like they were getting a lot from it... They wanted something extra to other students that weren’t doing it, so other halls that weren’t doing it. I think they wanted to feel like they were getting some more benefit than they actually did get.” [I, S10]
	Barrier (testing service)	“I think at first when they first sort of advertised the pilot scheme, it was very like glorified that it was like there’s literally no social distancing, no masks, like, it was all sort of focused on that, but then in reality when the actual sort of more detailed information came out once we’d all signed up to it, it was kind of not as it was advertised in a way” [FG2, S1] “I thought that with the relaxation there might be some activities put on for the whole Hall but that didn’t seem to happen at all” [FG4, S3] “I think that was why our participation got really low because all the students were like ‘we can’t be bothered, it’s not lived up to what it was supposed to be’” [FG5, S2]	False expectations	“It’s a shame that there wasn’t one positive case – it seems a really strange thing to say – but I think that that would have actually shown to people the way that we could then contain it, the fact that we would have been able to put in extra testing for that group of people, they wouldn’t have all had to self-isolate, the person who tested positive would have got the ability to isolate in the [alternative accommodation] and all things like that.” [I, S2]
	Barrier (peers)	“I think just as a generalisation of Halls I don’t think the social distancing mask thing within Halls, within bedrooms, that sort of thing in student only spaces, I don’t think it happens all that much, but it’s been the same the entire year, it’s not changed really with the pilot.” [FG4, S2] “So, when we went out into town for example, we’d stay as a group, instead of distancing, because of the pilot study” [FG1, S4] “it encouraged parties which would get out of hand, granted they were dealt with accordingly, but I do think it was seen by some as a reason to be reckless” [S]	Non-compliance with the rules (within and outside residence)	“I also think for the Hall staff that were involved in it, during those two weeks, I imagine it was really stressful for them because of the behaviour that they saw I guess they didn’t quite know what was going to come next, what was going to be round the corner and I imagine that can be quite daunting for them”. [I, S2]

Data source: *S* Survey (students), *FG* Focus group (students), *I* Interview (staff). This table is based on the COM-B framework (Michie et al., 2015): Capability (C), Opportunity (O), Motivation (M) and Behaviour (B)

#### Dose and timeliness

Tests were provided to students each week (2 per week) in line with planned delivery timing (scheduled days/times). Weekly ‘delivered’ dose and timeliness therefore aligned with the pre-determined plan (8 tests in total). However, it was not possible to assess whether overall ‘received’ dose was per protocol due to early termination of data collection around test compliance. Evaluation is therefore based on a reduced dose (3 tests) across a shorter intervention period (10 days) during which uptake and compliance data were collected.

The majority of students were satisfied with test pick-up and drop-off processes (84%, *n* = 121/144) and the communication around test results (96.6%, *n* = 140/144). Any dissatisfaction was generally due to a perception of poor communication around changed procedures, or perceived inappropriateness of drop-off timings due to academic commitments (this issue was identified early in the pilot and addressed on receipt of student feedback, with drop-off times extended). If a positive case was identified in a student’s hall during the pilot, all students in hall were required to take an additional test that week (surge testing). The vast majority, 97.1% (*n* = 136) of students participating in the pilot thought this plan was acceptable, although surge testing did not occur since there were no positive cases identified during the intervention period.

*“I thought it was a good thing because it spread so much in the October, November time so I felt like it definitely would have halted it.” [FG5, S3].*

Students who were identified as a close contact of a person who tested positive were required to test every day for 7 days. An estimated 96.4% (*n* = 134) felt that the 7-day contact testing frequency was acceptable: “*small price to pay for protecting others from covid*.” Students who were identified as a close contact of a person who tested positive did not need to isolate if their tests over the next 7 days were negative, and this was viewed to be acceptable (93.5%, *n* = 130/142). Only one (.7%) individual reported experiencing this process as they were identified as a close contact of a positive case. A further case (.7%) indicated they preferred ‘not to say’.

## Discussion

To our knowledge, this is the first process evaluation of a residence-based SARS-CoV-2 surveillance testing participation intervention in a university campus setting. The RB-TPP had high uptake and fidelity, resulted in a dramatic increase in the proportion of students engaging in surveillance testing (for the detection of SARS-CoV-2 RNA), and played a significant role in students’ mental wellbeing.

Uptake of the RB-TPP was high across both sites (98% participation of in-house students, 79% of listed occupants) with high engagement in testing (88% of programme participants testing, compared with 5% pre-intervention), albeit only half (46% of participants, 52% of testers) were fully compliant with the twice-weekly testing frequency during the data collection period. Based on the increase in testing uptake, staff and students viewed the RB-TPP to be successful despite not meeting the 90% target, and this pre-determined target was perceived by interviewees to be too high. Most of the student participants indicated they would take part again and would recommend the initiative to others. Students found the saliva test itself to be acceptable for regular testing; saliva testing for SARS-CoV-2 has been found to be a useful and acceptable tool for use in a mass screening context [37–39]. Although we did not explicitly assess students’ perceptions towards the reliability or safety of asymptomatic testing in this process evaluation, our finding that the tests were highly acceptable concurs with prior evidence showing that university students find saliva testing acceptable, with ease of donation and minimal invasiveness, and they are confident in the results [10, 16, 17].

The RB-TPP was largely implemented as planned. At the time of intervention delivery, there was a national surge in cases of the B.1.617.2 (Delta) variant of SARS-CoV-2. The Delta variant is associated with more severe disease than the previously dominant Alpha (B.1.1.7) variant as determined by twice the relative risk of hospital admission [40]; positive cases had been identified in the region. Due to this, NHS Test and Trace (national public health team) requested early termination of the relaxed social distancing rules in the RB-TPP, with students and staff to revert to adherence to the national behavioural rules applied at that critical time. Testing continued throughout the intervention period, albeit with a reduced data collection period relating to testing adherence in the RB-TPP. Since there were no reactive tests during the intervention period, surge testing and local contact tracing for positive cases were not required and so evaluation of these approaches ‘in action’ was not possible. However, students and staff held positive views towards the planned approaches.

University staff highlighted that using an accredited asymptomatic test would reduce the lag in isolating positive cases, through removal of the need to undertake a confirmatory test. This was subsequently resolved, since the university ATS used here was recommended for accreditation by UKAS (the National Accreditation Body for the UK) in July 2021, accelerating the containment process and removing the burden of confirmatory tests for national testing services. The original (pre-accreditation) process was that a positive PCR test with the ATS would lead to a *request* (with no legal grounds) for the individual to isolate and take a government Pillar 2 PCR confirmatory test. Therefore, the time from the initial saliva test in the RB-TPP to the result of the confirmatory test could be many days or even a week. After the recommendation for accreditation of the service was in place, the new approach considerably speeded up the process. Going forwards, a positive PCR test result from the ATS resulted in immediate notification of the individual who was then required to isolate by law (as the result was then equivalent to that of a government PCR test). In parallel, Public Health England (PHE) was notified of the positive result in order that further actions could be taken (i.e., identification and notification of close contacts), which happened the day after ATS sample provision.

Students were personally motivated to take part in the RB-TPP by the perceived safety of regular testing, societal responsibility to protect others, and a strong desire to socialise. These factors have previously been identified as important in SARS-CoV-2 testing uptake in higher education settings [16, 17]. Engagement in the programme was primarily facilitated by the positive impact of RB-TPP on students’ mental wellbeing, stemming from a reduced fear of self-isolation (regular testing of close contacts of positive cases, instead of self-isolation), social contact within their accommodation and a perceived return to normal university life. Sanctioning social contact was well received by the vast majority, despite perceptions from some students that the RB-TPP did not meet all their expectations with regards residence-wide social events. Motivation to participate was further enhanced through the involvement of student ambassadors to assist with communications and provide peer-to-peer support. Student ambassadors have been used successfully to raise health-awareness or advocate health screening programmes in educational settings (e.g., [41, 42]: COVID-19 vaccination [43];: COVID-19 communication and mitigation behaviours [44];: HPV vaccination [45];: flu vaccination). Our findings contribute to an emerging evidence-base advocating the role and impact of peer-to-peer student health ambassadors on campus to mitigate the spread of COVID-19 [46]. Views towards the provision of small incentives to maximise engagement in the programme were mixed. Incentives were perceived as either ‘motivating’, or ‘not enough’ (by students), ‘lacking’ or ‘inappropriate’ (by staff).

Adherence to testing was satisfactory. In the current study, conducted in 2021, we found that most students engaged in asymptomatic saliva testing (88%); this is comparable with a prior study conducted at the same institution in 2020, in which 89.2% of first year students completed one or more saliva samples during an intervention period [16]. In the current study, we found that 46% of participants were fully compliant with a pre-determined testing frequency; in 2020 we found that 47.7% of students completed a pre-determined testing protocol (albeit much longer - 12 weeks) [16]. There are few published reports of student ‘uptake’ and ‘compliance’ with SARS-CoV-2 testing, although this level of compliance appears to be higher than that reported in other university settings (albeit with variations in testing protocols). For example, lack of full compliance with a saliva-based SARS-CoV-2 testing protocol on a university campus in the United States of America was observed in 82.3% of participants (with reasons not assessed) [12]. Further, individual adherence was likely to be higher than documented, since students who left the residence during the study period or could not locate a test kit due to logistic issues were not excluded from test uptake figures. Adherence was facilitated by attitudes and views (about COVID-19 and the importance or protective behaviours), satisfaction with the level of social contact, and practical issues (the ease of the saliva test, the efficiency of the testing service and the convenience of testing within accommodation).

Testing adherence was hindered by changes in procedures or logistics, such as timings for sample drop-off (which were subsequently revised), and inconsistency in messages around rules and regulations delivered by different staff groups and student representatives. One barrier to intervention success was complacent attitudes and non-compliance with the rules in a minority, which was challenging for staff and frustrating for adhering students. Complacent attitudes and misbehaviour of some students caused frustration among those conforming to the rules. It was not possible to determine whether (and how) students were mixing with others outside of the residences, or in the general community and so this cannot be ruled out. However, it should be borne in mind that the purpose of the RB-TPP was to provide an asymptomatic testing service which would allow for the identification of people who were asymptomatic (including pre-symptomatic) or who had the symptoms that were not being promoted in national guidance at the time (e.g., headache, sore throat etc). The sensitivity of Quantitative Reverse Transcription PCR (RT-qPCR) meant the aim was for early detection, and hence removal of people testing positive from the circulating population, thereby reducing onward transmission.

Non-compliance with behavioural regulations (e.g., face coverings, social distancing) was largely due to misunderstanding (of students and some staff) about what was allowed and where, and frequent changes or perceived inconsistency in messaging. Although central briefings to students were delivered by email and contained accurate and up-to-date information, the rapidly changing external context of the pandemic meant that communications and updates were very frequent, and written communication was often perceived to be lengthy and complex in nature. Feedback from students indicated that not all had accessed and read the written communications in a timely way. Concurrently, staff in student-facing roles were challenged to continually communicate updates, verbally, in a regularly changing context and among students with varying levels of familiarity with new processes and procedures. Studies of health messaging during previous pandemics (e.g., [47]: H1N1) have identified the challenges of communicating effectively to staff and students about the spread of viruses without inciting unnecessary fear or promoting complacency. Further, communications need to account for known variability in health literacy in student populations [48], and empathy in messaging is critical but often overlooked in a pandemic situation [49].

The prevalence of mental health concerns among students at this point in the pandemic should not be underestimated. Our qualitative findings highlight the widespread belief that relaxed social distancing within the residence was beneficial for students’ mental well-being; some students viewed this social contact as essential for their mental health. Nevertheless, in our survey, all students reported signs of anxiety and almost half our sample (46%) had clinically relevant anxiety levels (moderate to severe score on the GAD-7). The negative impact of the COVID-19 pandemic on university students’ mental well-being is already established [17, 50–53], is associated with increased social isolation [54] and is likely to have long-term consequences on students’ health and education [55]. This highlights the importance of initiatives that create opportunities for safe social contact during the pandemic. Social interaction was deemed to be exceptionally important for mental health by this student group and there was an overwhelming perception that the benefits of social interaction to mental health outweighed the risk of virus transmission, which they perceived to be low. Therefore, efforts to engage students in COVID-19 mitigation initiatives that provide socialising opportunities and a perceived return to a more ‘normal university experience’ may be more successful than those focusing on testing uptake alone.

Our results will help to inform whether, and how, asymptomatic testing could be implemented in residences at other campus-based university settings as part of COVID-19 outbreak prevention and management approaches in higher education environments. Although the intervention was shorter than planned, findings support the premise that that residence-based high-frequency repeated testing may be an effective strategy for COVID-19 mitigation. The RB-TPP approach was perceived by students and staff to be acceptable, increased perceptions of safety on campus and assisted in normalisation of university life with benefits for mental well-being during an extended pandemic. This process evaluation supports the implementation of such schemes, but future success relies on the necessary infrastructure or funding for implementation, expectation checks with students, and consistency of messaging relating to changes in processes and behavioural expectations. Key findings and recommendations are shown in Table 5.

**Table 5:** Key findings and recommendations

***Key findings***	
• Offering COVID-19 tests in university residences is viewed positively, with broad uptake and reach.	
• Testing engagement is high (88%), compliance with twice-weekly testing is lower (46%).	
• The concept of surge testing is seen to be an acceptable outbreak mitigation strategy.	
• Regularly testing contacts of positive cases is perceived to be preferable to self-isolation.	
• The concept of surge testing is seen to be an acceptable outbreak mitigation strategy.	
• Relaxed social restrictions improve student satisfaction and mental health.	
• Most students are compliant with residence-based COVID-19 social regulations but those who are not create challenges and stressors for peers and staff.	
• Responding to the changing landscape of a pandemic is challenging for staff.	
***Key recommendations to maximise uptake, adherence and compliance***	
• Written communications for students should be briefer, empathetic, positive and persuasive but avoid punitive tone.	
• Students require clear and specific rules outlining expectations around social distancing, mask wearing and socialising, with explanations for changes.	
• Communications should be delivered consistently across all staff groups to avoid mixed messages.	
• Timings for kit collection and sample drop-off need to be accessible around meals and academic commitments.	
• Involving students in programme planning is essential to ensure procedures and communications match the needs of the student population.	
• Involving staff with student-facing roles in programme planning is essential to establish clear lines of communication and to ensure procedures are practical and achievable.	
• Student ambassadors are a useful mechanism for provision of peer-to-peer support and assistance with communications.	
• Regular updates on testing uptake rates are valued by and motivate students.	
• Normalising residence-based testing will be important for future uptake of surveillance testing during a pandemic.	

### Study limitations

Log file analysis for identification of multiple survey entries was not used; Internet Protocol (IP) addresses were not available to protect confidentiality. The inclusion of monetary incentive for completion of the survey may have introduced bias into the sample. However, this is unlikely since the financial value of the incentive was very low (£5), and prior studies have not found any significant differences on response completeness between those who received an incentive offer and those who did not [56]. The survey response rate was low, although respondents were broadly representative of the wider pool of students registered as living at the two participating sites. The views of non-participants in the programme were invited but are under-represented.

### Reflection on rapid process evaluation approaches

The process evaluation was designed in alignment with a pre-determined logic model, which assumes linear and predictive pathways. However, our findings demonstrate that even over a 4-week period, participants (students, service providers) may adapt (intentionally or unintentionally) as they respond to feedback (e.g., from university students or staff, and local public health teams) and contextual changes (e.g., escalation of the Delta variant, changes in national guidelines). The context of rapidly changing global circumstances required immediate responsiveness in local outbreak prevention and management approaches. Our study went beyond adherence or non-adherence to the implementation plan and recognised the reasons for in-situ changes as they occurred, allowing for process evaluation to feedback into the operationalisation of the service. As such, for a process evaluation conducted alongside the implementation of an intervention within a complex organisational system, in the context of a pandemic we would advocate for a more developmental approach requiring an emergent perspective [57, 58].

## Conclusions

To our knowledge, this study is the first mixed-methods process evaluation conducted alongside a university residence-based asymptomatic SARS-CoV-2 testing intervention, during the global COVID-19 pandemic. The RB-TPP intervention for students in university residences increased testing behaviour and improved students’ mental wellbeing. It was adequately delivered, well-received and could be implemented more widely with some modifications to optimise future delivery.

## Supplementary Information

**Additional file 1. **Explanation of terms.

**Additional file 2. **Consolidated criteria for reporting qualitative studies (COREQ): 32-item checklist.

**Additional file 3. **The TIDieR (Template for Intervention Description and Replication) Checklist*.

**Additional file 4. **Testing Participation Pilot: Student Survey.

**Additional file 5. **Recruitment announcement and Question Guides.

**Additional file 6. **Student health and exposure to COVID-19.

## Data Availability

The datasets used and/or analysed during the current study are available from the corresponding author on reasonable request.

## Abbreviations

ATS: Asymptomatic Testing Service
COM-B: Capability, Opportunity, Motivation – Behaviour
PCR: Polymerase Chain Reaction
PPIE: Participant and Public Involvement and Engagement
RT-qPCR: Reverse transcription polymerase chain reaction
RNA: Ribonucleic acid
RB-TPP: Residence-Based Testing Participation Pilot
SARS-CoV-2: SARS Coronavirus 2
UK: United Kingdom
