# Supplementary material for: Mixed-methods process evaluation of a residence-based SARS-CoV-2 testing participation pilot on a UK university campus during the COVID-19 pandemic
Source: BMC Public Health. 2022 Aug 2;22:1470. doi: 10.1186/s12889-022-13792-8 (PMC9343222; doi:10.1186/s12889-022-13792-8)
Supplement: Supplementary file 1 — Additional file 1. Explanation of terms. [file 12889_2022_13792_MOESM1_ESM.pdf]

Additional file 1: Explanation of terms

Term	Explanation
Programme title	Mixed-methods process evaluation of a Residence-Based SARS-CoV-2 Testing Participation Pilot on a UK university campus during the COVID-19 pandemic
Short title	RB-TPP
Testing	This consists of asymptomatic PCR testing at two student residences on a university campus.
PCR Test	Saliva test for antigens to detect infection with SARS-CoV-2.
Behavioural elements	This consists of social distancing, wearing face masks, hand hygiene.
RB-TPP Programme components	The RB-TPP programme has 3 main components: 1. Asymptomatic SARS-Cov2 testing; 2. Relaxed behavioural restrictions within residences; 3. Student ambassadors to support communications and messaging.
RB-TPP Participants	These are:<br>• Students who opted into the RB-TPP at one of the two host sites and took part in any of the programme components.<br>• Students who opted out of the RB-TPP and was not living in the residence during the intervention period.
Student Ambassadors	This is a group of trained/supported students who supported *engagement with* and *adherence to* the RB-TPP. This was achieved by motivating and encouraging students in each intervention site to engage in asymptomatic testing, adhere to 'rules' set within the intervention sites, and assist in the communication of messages to students across the two sites.
Fidelity	The extent to which the RB-TPP is delivered as planned at each site.<br>*Reach* (the proportion of the target group who participated in RB-TPP and their socio-demographic characteristics), *Dose* and *Timeliness* (of the intervention delivered) and *Adherence/Compliance* (of students to the minimum programme requirements).
Essential elements	Aspects of the RB-TPP which are hypothesised to be critical to the effectiveness of the programme and must therefore be delivered. Figure 1. shows the essential elements of RB-TPP.
Participating agencies	Participating agencies are the key sites (two student residences on a single university campus) involved in the programme delivery or implementation, selected from a larger sample frame (all student residents across the university).
Liaison Person	The operations lead for the RB-TPP acted as intermediary and facilitator for the RB-TPP process evaluation. This liaison person was briefed on the process evaluation and remained in regular contact with the project team.
Service providers	Service providers are referred to in three groups:<br>• Staff with a role in intervention design<br>• Staff with a role in intervention delivery - operations or oversight - including working with agencies to tailor the programme to changing circumstances or arising needs.<br>• Staff with a role in student pastoral care / welfare support.
