# Supplementary material for: Mixed-methods process evaluation of a residence-based SARS-CoV-2 testing participation pilot on a UK university campus during the COVID-19 pandemic
Source: BMC Public Health. 2022 Aug 2;22:1470. doi: 10.1186/s12889-022-13792-8 (PMC9343222; doi:10.1186/s12889-022-13792-8)
Supplement: Supplementary file 2 — Additional file 2. Consolidated criteria for reporting qualitative studies (COREQ): 32-item checklist. [file 12889_2022_13792_MOESM2_ESM.pdf]

Additional file 2. Consolidated criteria for reporting qualitative studies (COREQ): 32-item checklist

Topic and Item No.	Guide Questions/Description	Response
**Domain 1: Research team and reflexivity**		
Personal Characteristics		
1. Interviewer/facilitator	Which author/s conducted the interview or focus group?	Sophie Carlisle and Lauren Fothergill
2. Credentials	What were the researcher's credentials? E.g. PhD, MD	PhD researchers
3. Occupation	What was their occupation at the time of the study?	Research Associates (Health Sciences)
4. Gender	Was the researcher male or female?	Both female
5. Experience and training	What experience or training did the researcher have?	Mixed methods researchers, experienced interviewers and focus group moderators, GCP trained
Relationship with participants		
6. Relationship established	Was a relationship established prior to study commencement?	Researchers met the participants during recruitment
7. Participant knowledge of the interviewer	What did the participants know about the researcher? e.g. personal goals, reasons for doing the research	Participants knew that the interviewers were university researchers. They knew that both interviewers were not involved in the delivery of the asymptomatic testing service or RB-TPP scheme.
8. Interviewer characteristics	What characteristics were reported about the interviewer/facilitator? e.g. Bias, assumptions, reasons and interests in the research topic	Participants knew that SC and LF were interested in the experiences of students during the COVID-19 pandemic.
**Domain 2: Study design**		
Theoretical framework		
9. Methodological orientation and theory	What methodological orientation was stated to underpin the study? e.g. grounded theory, discourse analysis, ethnography, phenomenology, content analysis	Thematic analysis
Participant selection		
10. Sampling	How were participants selected? e.g. purposive, convenience, consecutive, snowball	Students: convenience sample<br>Staff: purposive sample
11. Method of approach	How were participants approached? e.g. face-to-face, telephone, mail, email	Participants were approached and recruited by email
12. Sample size	How many participants were in the study?	Student focus groups: 30<br>Staff Interviews: 13
13. Non-participation	How many people refused to participate or dropped out? Reasons?	No participants actively withdrew.
Setting		
14. Setting of data collection	Where was the data collected? e.g. home, clinic, workplace	Data were collected online (video-conferencing platform)
15. Presence of non-participants	Was anyone else present besides the participants and researchers?	Two researchers were present at focus groups. There was one administrator present at one focus

		group to act as scribe (in order to rapidly feedback key findings to the oversight team).
16. Description of sample	What are the important characteristics of the sample? e.g. demographic data, date	Students: Residence (site 1 or 2). Staff: Job role.
Data collection		
17. Interview guide	Were questions, prompts, guides provided by the authors? Was it pilot tested?	Yes. The questioning guide was pilot tested with students who were not participants in this study.
18. Repeat interviews	Were repeat interviews carried out? If yes, how many?	No repeat interviews.
19. Audio/visual recording	Did the research use audio or visual recording to collect the data?	Interviews and focus groups were audio-recorded using a video-conferencing platform. Interviewers kept cameras on to assist with establishing rapport.
20. Field notes	Were field notes made during and/or after the interview or focus group?	Yes.
21. Duration	What was the duration of the interviews or focus group?	Staff Interviews: approx. 60 mins Student Focus groups: approx. 60-90 mins.
22. Data saturation	Was data saturation discussed?	Yes.
23. Transcripts returned	Were transcripts returned to participants for comment and/or correction?	Yes - a sub-sample.
**Domain 3: analysis and findings**		
Data analysis		
24. Number of data coders	How many data coders coded the data?	Two.
25. Description of the coding tree	Did authors provide a description of the coding tree?	No, however initial coding was informed by the interview guide, and coding was continuously refined.
26. Derivation of themes	Were themes identified in advance or derived from the data?	These were derived from the data.
27. Software	What software, if applicable, was used to manage the data?	N'Vivo
28. Participant checking	Did participants provide feedback on the findings?	Yes.
Reporting		
29. Quotations presented	Were participant quotations presented to illustrate the themes / findings? Was each quotation identified? e.g. participant number	Yes.
30. Data and findings consistent	Was there consistency between the data presented and the findings?	Yes.
31. Clarity of major themes	Were major themes clearly presented in the findings?	Yes.
32. Clarity of minor themes	Is there a description of diverse cases or discussion of minor themes?	Yes.
