# Supplementary material for: Mixed-methods process evaluation of a residence-based SARS-CoV-2 testing participation pilot on a UK university campus during the COVID-19 pandemic
Source: BMC Public Health. 2022 Aug 2;22:1470. doi: 10.1186/s12889-022-13792-8 (PMC9343222; doi:10.1186/s12889-022-13792-8)
Supplement: Supplementary file 4 — Additional file 4. Testing Participation Pilot: Student Survey. [file 12889_2022_13792_MOESM4_ESM.pdf]

Testing Participation Pilot: Student Survey

Page 1: About this Survey

We would like to know more about your views towards the COVID-19 testing pilot in halls. This will help to inform future plans for containing the spread of COVID-19 in higher education settings.

Page 2: About You

1. How old are you? (Please enter a whole number)

2. What is your gender identity?

- Male
- Female
- Other
- Prefer not to say

2.a. If 'other', please specify:

3. What is your ethnicity?

- White
- Mixed
- Asian or Asian British
- Black or Black British
- Middle Eastern or Middle Eastern British
- Other ethnic group
- Prefer not to say

3.a. If 'other ethnic group', please specify:

3.b. Are you:

- British
- Irish
- Any other white background

3.c. Are you:

- White and black Caribbean
- White and black African
- White and Asian
- Other mixed background

3.d. Are you:

- Chinese
- Indian
- Pakistani
- Bangladeshi
- Other Asian background

3.e. Are you:

- Caribbean
- African
- Any other Black background

3.f. Are you:

- Arab
- Turkish
- Any other Middle Eastern background

4. Do you know anyone that (select **all** that apply):

- [ ] Had (or think they have had) COVID-19
- [ ] Had to be in hospital with COVID-19
- [ ] Died from COVID-19
- [ ] None of the above

5. Are you an international student (i.e., not from the UK)?

- Yes, I'm from the EU
- Yes, I'm from outside the EU
- No, I'm from the UK

6. What stage of the study are you at?

- Foundation course/ certificate
- 1st year of undergraduate study
- 2nd year of undergraduate study
- 3rd year of undergraduate study
- 4th year of undergraduate study
- 5th year or higher of undergraduate study
- Postgraduate

7. Do you have caring responsibilities?

- Yes
- No
- Prefer not to say

8. Do you have a history of anxiety, depression or any other mental health issue for which you have received treatment in the past?

- Yes
- No
- Prefer not to say

9. Do you have an existing physical health issue that you think could affect your risk of getting COVID-19?

- Yes
- No
- Prefer not to say

10. Where are you currently living during term-time?

- Halls of residence
- Temporary alternative accommodation (e.g., another hall or Orchard Hotel)

10.a. If hall of residence, which are you living in?

- Albion House

- Ancaster Hall
- Beeston Hall
- Bonington Student Village
- Broadgate Park
- Cavendish Hall
- Cloister House
- Cripps Hall
- Dagfa House
- Derby Hall
- Florence Boot Hall
- Hugh Stewart Hall
- Jubilee Hall
- Lenton and Wortley Hall
- Lincoln Medical School students
- Melton Hall
- Newark Hall
- Nottingham Two
- Raleigh Park
- Riverside Point
- Royal Derby Hospital Medical School students
- Rutland Hall
- Sherwood Hall
- Southwell Hall
- St Peter's Court
- The Vantage
- Varsity
- Willoughby Hall

10.b. If temporary, for what reason are you in alternative accommodation?

- Chose not to take part in the testing participation pilot
- Tested positive for COVID-19 during the testing participation pilot
- Other reason

10.b.i. If 'other reason', please specify:

11. Are you doing paid or voluntary work during term-time?

- Yes
- No
- Prefer not to say

12. Does this work bring you into close contact with others? By close contact we mean people who are within 2 metres of you.

- Yes
- No

13. Are you currently fulfilling any of the government's identified 'keyworker' roles?

- Health, social care, or relevant related support worker
- Teacher or childcare worker, still travelling in to work
- Transport worker, still travelling in to work
- Food chain worker (production, sales, delivery)
- Key public services worker (e.g., justice staff, religious staff, public services journalist or mortuary worker)
- Local or national government worker delivering essential public services
- Utility worker (e.g., energy, sewerage, postal services)
- Public safety or national security worker
- Worker involved in medicines or protective equipment production or distribution
- Other key worker role not listed
- None of these

14. Have you tested positive for COVID-19 any time during the pandemic, *before the testing participation pilot?*

- Yes, once
- Yes, more than once
- No
- Prefer not to say

15. Have you self-isolated at any time during the pandemic, *before the testing participation pilot*?

- Yes, once
- Yes, more than once
- No

- Prefer not to say

16. Have you been shielding at any time during the pandemic? By this we mean being advised to protect yourself from COVID-19 due to being considered high-risk.

- Yes
- No
- Prefer not to say

Page 3: Your Participation in the Pilot

17. Did you take part in the testing participation pilot?

- Yes
- No

18. What were your main reasons for taking part? Please select **all** relevent options.

- To protect yourself
- To protect my family
- To protect local communities
- To reassure myself about my health status
- To reassure my family about my health status
- To contribute to national efforts to contain COVID-19
- Helping to keep campus safe for everyone
- Learning something new about COVID-19 itself
- Learning something new about COVID-19 testing
- Getting to know other students better
- Getting to know university staff better
- Being involved in COVID-19 research
- Having pride in my University
- Other (you will be asked to specify)

18.a. If 'other', please specify:

19. How did you feel about the information you received about the pilot?

- Extremely satisfied
- Somewhat satisfied
- Somewhat unsatisfied
- Extremely unsatisfied

19.a. Tell us why:

20. Did you take ALL of the saliva based COVID-19 tests you were offered over the *last 4 weeks*?

- Yes
- No

20.a. If no, tell us why:

21. How many tests did you take in total over the 4-week pilot?

- None
- 1
- 2
- 3
- 4
- 5
- 6
- 7
- 8
- 9
- 10
- 11
- 12
- 13
- 14
- 15
- 16
- 17
- 18
- 19
- 20
- 21
- 22

- 23
- 24
- 25
- 26
- 27
- 28

22. How did you feel about the physical process of undertaking the saliva test?

- Satisfied
- Not satisfied

23. How did you feel about the pick-up and drop-off processes?

- Satisfied
- Not satisfied

24. How did you feel about the communication of test results?

- Satisfied
- Not satisfied

25. How confident were you in the results of your saliva tests?

- Extremely confident
- Somewhat confident
- Somewhat unconfident
- Extremely unconfident

26. How did you feel about being able to interact with other people in your hall during this time?

- Extremely satisfied
- Somewhat satisfied
- Somewhat dissatisfied
- Extremely dissatisfied

26.a. Tell us why:

27. Did you wear a face covering in communal areas of the hall (except in designated eating areas and student bedrooms)?

- Yes, always
- Yes, sometimes
- No

27.a. Tell us why:

28. How did you feel about relaxed social distancing between students in the hall?

- Acceptable to me
- Not acceptable to me

28.a. Tell us why:

29. Did you maintain a 2-metre social distance when interacting with staff?

- Yes, always
- Yes, sometimes
- No

29.a. Tell us why:

30. Did you adhere to social distancing and all other COVID-19 security rules outside of the hall environment?

- ○ Yes
- ○ No
- ○ N/A - I didn't leave the hall during this time

30.a. Tell us more:

[ ]

31. Did you have to self-isolate at any time during the testing pilot?

- ○ Yes, once
- ○ Yes, more than once
- ○ No

31.a. Why did you have to self-isolate?

- ○ I experienced COVID-19 symptoms but didn't take a test
- ○ I tested positive for COVID-19
- ○ Someone in my accommodation tested positive for COVID-19
- ○ Someone in my accommodation had symptoms but did not take a test
- ○ I came into contact with someone outside my accommodation that tested positive for COVID-19
- ○ Other (e.g. return from travel to high-risk area)

31.b. Which statement best describes your experience of self-isolation?

- ○ I stayed in my own room and had no contact with anyone else.
- ○ I mostly stayed in my own rooms but had some contact with other people in my accommodation (e.g. eating meals together)
- ○ I stayed in my accommodation but did not stay in my room (e.g. had regular contact with people in my accommodation but did not have contact with anyone outside my accommodation)
- ○ I did not self-isolate (e.g. I had contact with people outside my accommodation)

32. Were you satisfied with the University's support during this period of self-isolation?

- ○ Extremely satisfied
- ○ Somewhat satisfied
- ○ Somewhat unsatisfied
- ○ Extremely unsatisfied

33. If a positive case was identified in hall during the pilot, all students in hall were required to take an additional test that week. How did you feel about this process?

- Acceptable to me
- Not acceptable to me

33.a. Tell us why:

34. Was there a positive case identified in your hall during the pilot?

- Yes
- No

34.a. If yes, did you take the additional test that week?

- Yes
- No

34.a.i. If no, why not?

35. Students who were identified as a close contact of a person who tested positive were required to test every day for 7 days. How did you feel about this testing frequency?

- Acceptable to me
- Not acceptable to me

35.a. Tell us why:

36. Students who were identified as a close contact of a person who tested positive did not need to self-isolate if their tests over the next 7 days were negative. How did you feel about this plan?

- Acceptable to me
- Not acceptable to me

36.a. Tell us why:

[ ]

37. Were you identified as a close contact of a positive case during the pilot?

- Yes
- No
- Prefer not to say

37.a. If yes, did you take **all** 7 tests for that week?

- Yes
- No

37.a.i. Tell us why:

[ ]

38. Did you test positive for COVID-19 at any time during the testing participation pilot?

- Yes
- No
- Prefer not to say

38.a. Were your contacts traced by the University contact tracing team?

- Yes
- No

38.b. How efficient was this process?

- Very efficient
- Somewhat efficient
- Somewhat inefficient
- Very inefficient

38.b.i. Tell us why:

38.c. Did you self-isolate after your positive saliva test result?

- Yes, for some of the time
- Yes, for part of the time
- No, I did not

38.c.i. Tell us why:

38.d. Did you take the confirmatory Pillar II test after your positive saliva test?

- Yes
- No
- Prefer not to say

38.e. Did you advise the Asymptomatic Testing Service of the results of your Pillar II test?

- Yes
- No
- Prefer not to say

39. Would you take part in a similar COVID-19 testing scheme in your accommodation in the future?

- Yes
- No
- Unsure

40. Would you recommend participation in this sort of testing scheme to your peers?

- Yes
- No
- Unsure

41. Is there anything about the testing participation pilot that has been difficult or less positive?

42. Is there anything else you would like to tell us about the testing participation pilot?

Page 4: Your Views and Feelings

43. On scale of 1-10, **BEFORE THE TESTING PARTICIPATION PILOT,** what did you believe your risk of getting COVID-19 was?

- 1 - I didn't think I would get it
- 2
- 3
- 4
- 5
- 6
- 7
- 8
- 9
- 10 - I knew I would most certainly get it

44. On scale of 1-10, **AFTER THE TESTING PARTICIPATION PILOT,** what do you currently believe your risk of getting COVID-19 is?

- 1 - I don't think I will get it
- 2
- 3
- 4
- 5
- 6
- 7
- 8
- 9
- 10 - I know I will most certainly get it

45. On a scale of 1-10, how important is:

Please don't select more than 1 answer(s) per row.

	1 - not at all important	2	3	4	5	6	7	8	9	10 - extremely important
... social distancing in controlling the risk of COVID-19?	☐	☐	☐	☐	☐	☐	☐	☐	☐	☐

... wearing face coverings in controlling the risk of COVID-19?	☐	☐	☐	☐	☐	☐	☐	☐	☐	☐
... handwashing in controlling the risk of COVID-19	☐	☐	☐	☐	☐	☐	☐	☐	☐	☐
... self-isolation (when advised to do so) in controlling the risk of COVID-19	☐	☐	☐	☐	☐	☐	☐	☐	☐	☐

46. Please read the following statements carefully and then select the one which best describes how you have been feeling over the *past 2 weeks*.

- ○ I do not worry about getting COVID-19
- ○ I occasionally worry about getting COVID-19
- ○ I spend much of my time worrying about getting COVID-19
- ○ I spend most of my time worrying about getting COVID-19

47. Please read the following statements carefully and then select the one which best describes how you have been feeling over the *past 2 weeks.*

- ○ I do not worry about my close friend(s)/relatives getting COVID-19
- ○ I occasionally worry about my close friend(s)/relatives getting COVID-19
- ○ I spend much of my time worrying about my close friend(s)/relatives getting COVID-19
- ○ I spend most of my time worrying about my close friend(s)/relatives getting COVID-19

48. Over the last 2 weeks, how often have you been bothered by the following?

Please don't select more than 1 answer(s) per row.

	Not al all	Several days	More than half the days	Nearly every day
Feeling nervous, anxious or on edge	☐	☐	☐	☐

Not being able to stop or control worrying	☐	☐	☐	☐
Worrying too much about different things	☐	☐	☐	☐
Trouble relaxing	☐	☐	☐	☐
Being so restless that it is hard to sit still	☐	☐	☐	☐
Becoming easily annoyed or irritable	☐	☐	☐	☐
Feeling afraid as if something awful might happen	☐	☐	☐	☐

Page 5: Follow-up

49. Would you be willing to share your views by taking part in a confidential discussion group?

- ○ Yes, I consent to take part
- ○ No, I don't want to take part

49.a. If yes, please provide a valid email address here. This is just so that we can contact you to arrange this, but your email address will be separated from your survey responses. Please enter a valid University email address:

Page 6: Final page
