# Supplementary material for: Mixed-methods process evaluation of a residence-based SARS-CoV-2 testing participation pilot on a UK university campus during the COVID-19 pandemic
Source: BMC Public Health. 2022 Aug 2;22:1470. doi: 10.1186/s12889-022-13792-8 (PMC9343222; doi:10.1186/s12889-022-13792-8)
Supplement: Supplementary file 5 — Additional file 5. Recruitment announcement and Question Guides. [file 12889_2022_13792_MOESM5_ESM.pdf]

Additional file 5. Recruitment announcement and Question Guides

[1] Survey and Focus Group Announcement

Dear Student,

We are conducting an evaluation of the University's Testing Participation Pilot in halls of residence.

We are interested in hearing the views of students who took part, as well as those who didn't. Your views are very important to us and will help to inform services and support for students in the future.

The focus group will last about one hour and will be conducted online via Microsoft Teams. The discussions are confidential, and you would not be identified individually in any reports.

If you take part, you will receive a £20 gift voucher by way of thanks for your time.

To join a focus group, please:

1. Read the attached information sheet.
2. Provide your consent to participate at the following link: [insert event booking link]
3. Sign up to just ONE of these groups via the Teams link:

[insert event booking links for 7 focus groups with dates / times occurring at the end of week 2]

You are also invited to complete a survey online, and if you choose to complete it you are entitled to receive a £5 voucher (you can provide your email address within the survey, and your email will be separated from your survey responses).

The survey can be completed here: [JISC Online Survey link]

We would encourage you to complete the survey AND attend a focus group if possible.

If you have any questions about the study, please contact project researcher: [name / email]

Thank you for your time.
The Evaluation Team.

[2] Student focus group questions

To establish participants' views on key aspects of the programme:

1. Audio-Recorded with consent.
2. Consent to participate.
3. Collect demographics (taken part or not, age, gender, home/international student)
4. Question set and prompts (adjust according to whether they took part or not)

a) General views
Towards being at University at this point in time, towards living in halls (+ve or -ve)
Towards provision of testing at the University more broadly (feel more or less at risk?).
What were your main reasons for taking part in this pilot? Or reason for decline?

b) Testing Participation Pilot Processes

i) Twice-weekly testing for all students in the pilot Halls]

- Did you feel you had enough information about the testing and the processes for this scheme? What are your views on how and when information was *communicated*?
- How acceptable was the *frequency* of the testing? *(2 times per week, at least 2 days apart).*
- How acceptable was the *process* of the saliva test itself *(in terms of ease of testing, convenience, discomfort or burden).*
- How acceptable were the *logistics* of the testing (*in terms of receiving test kits and dropping off samples)*
- If you didn't take part, what is your view towards the Testing Participation Pilot?

ii) Views towards allowing greater social interaction *within* the Hall

- What are your views towards this and why? How did you feel about the relaxed social interaction approach in halls? (*compared with enforcement of social distancing in halls, and the previous approach focused on enforcement of household-only mixing).*
- What went well or less well? *(e.g., social contact, activities, perceptions of risk/exposure, interaction in shared facilities, catering)*
- Were students wearing masks in communal spaces and when interacting with staff?
- If you didn't take part, how do you feel about Testing in halls to allow greater social interaction between students?

iii) Any impacts of the approach on behaviours?

- Did the approach in halls change any behaviours outside halls? Were students still complying with social distancing outside the hall during this time? *(elsewhere in the University, off-campus).*
- Did it alter students' behaviours outside of the hall with regards masks and social distancing?
- If you didn't take part, did you hear of, or notice any changes in behaviours of students that did?

iv) Surge testing and enhanced contact tracing

- Plans include testing all hall residents if there is a positive case in the hall. What is your view on this? Has this procedure occurred If so, how did it go?
- Did you have any liaison with the special contact tracing team? What is your view on the contact tracing procedure during this time? (enhanced contact tracing regime)
- If you didn't take part, what are your views towards testing everyone in a hall if there's a positive case, and towards the University supporting contact tracing processes more locally?

v) Increased support for isolation and positive cases

- What is your view towards the support offered to students who had to self-isolate during this period (this may be due to testing positive or being a contact of someone who tested positive) or alternatively, the plans to, if it didn't happen. For example, support provided by the University – testing team, welfare support, peer support, transfer to alternative accommodation etc. Did you have to transfer to alternative accommodation during this time and how was that?

c) Anything else you would like to tell us? Do you have any recommendations for the future? More of these schemes in halls? Or other ways to encourage uptake of testing among students going forwards?

[3] Testing Champions focus group questions

To establish participants' views on key aspects of the programme:

1. Audio-Recorded with consent.
2. Consent to participate.
3. Collect demographics (taken part or not, age, gender, home/international student)
4. Question set and prompts (adjust according to whether they took part or not)

a) General views
Towards being at University at this point in time, towards living in halls (+ve or -ve)
Towards provision of testing at the University more broadly (feel more or less at risk?).
What were your main reasons for taking part in this pilot? Or reason for decline?

b) Testing participation processes and impacts
What are your views towards:

- Testing to allow greater social interaction in halls
- Regular testing - Twice weekly and surge testing if there is a positive case
- Were the students generally compliant with the testing? And with regulations such as mask wearing?
- Do you think the pilot impact on students' wellbeing or behaviours in any way? (+ve or -ve)
- What was the benefit of the Testing champions role (peer to peer encouragement)?
- Did it help encourage testing uptake?
- Is there anything that worked particularly well, or less well, about the pilot?
- Is there anything that worked particularly well, or less well, about the testing champions role?
- Anything else you would like to tell us? Recommendations for the future? More of these schemes in halls? Or other ways to encourage uptake of testing among students going forwards?

[4] Staff interview questions
(appropriateness, effectiveness, efficiency)

1. To what extent does mass testing help in containing the spread of COVID-19 in universities? What impact (if any) do you think the Testing Participation scheme will have on containing the spread of COVID-19 (in the short-term, medium-term, and longer-term)?

2. What has the impact of the scheme been on students *(e.g., personal risk and wellbeing, satisfaction, social behaviours, testing processes, identifying cases, wellbeing etc)*, and in any particular groups?

3. What has the impact of the scheme been on staff *(e.g., personal risk and wellbeing, satisfaction, resources etc)* and in any particular groups?

4. What has worked well? What were the particular features of the scheme and the context that were most helpful / made a positive difference? *(e.g., communications, process, test type, logistics, social aspects, surge testing, enhanced contact tracing, managing positive cases, isolation support).*

5. What has worked less well? What were the particular features of the scheme and the context that were least helpful / had a negative impact? (*e.g., communications, process, test type, logistics, social aspects, surge testing, enhanced contact tracing, managing positive cases, isolation support).*

6. Was there anything else that influenced *how* the testing scheme was implemented and the outcome *(e.g., social factors, government policy, new guidance, incidents)*

7. What is your view on using resources in this way?

8. Anything else you would like to tell us? Recommendations for the future? More of these schemes in halls? Or other ways to encourage uptake of testing among students going forwards?
