# Supplementary material for: Mixed-methods process evaluation of a residence-based SARS-CoV-2 testing participation pilot on a UK university campus during the COVID-19 pandemic
Source: BMC Public Health. 2022 Aug 2;22:1470. doi: 10.1186/s12889-022-13792-8 (PMC9343222; doi:10.1186/s12889-022-13792-8)
Supplement: Supplementary file 6 — Additional file 6. Student health and exposure to COVID-19. [file 12889_2022_13792_MOESM6_ESM.pdf]

**Additional file 6.** Student health and exposure to COVID-19.

| | n(%) | | |
|---|---|---|---|
| | Total sample | P | Non-P |
| **Tested positive for COVID-19** | N=151 | n=144 | n=7 |
| Yes, once | 48 (31.8) | 48 (33.3) | 0 |
| Yes, more than once | 6 (4%) | 6 (4.2) | 0 |
| No | 97 (64.2) | 90 (62.5) | 7 (100) |
| Prefer not to say | 0 | 0 | 0 |
| **Need to self-isolate any time prior to pilot** | N=151 | n=144 | n=7 |
| Yes, once | 84 (55.6) | 82 (56.9) | 2 (28.6) |
| Yes, more than once | 42 (27.6) | 42 (29.2) | 0 |
| No | 24 (15.9) | 19 (13.2) | 5 (71.4) |
| Prefer not to say | 1 (.7) | 1 (.7) | 0 |
| **Need to shield at any time prior to pilot** | N=151 | n=144 | n=7 |
| Yes | 7 (4.6%) | 7 (4.9) | 0 |
| No | 142 (94%) | 135 (93.8) | 7 (100) |
| Prefer not to say | 2 (1.3%) | 2 (1.4) | 0 |
| **Prior history of anxiety, depression, or other mental health challenge** | N=150 | n=143 | n=7 |
| Yes | 29 (19.3) | 28 (19.6) | 1 (14.3) |
| No | 112 (74.7) | 108 (75.5) | 4 (57.1) |
| Prefer not to say | 9 (6.0) | 7 (4.9) | 2 (28.6) |
| **Existing physical health issue that could affect your risk of getting COVID-19** | N=151 | n=144 | n=7 |
| Yes | 7 (4.6) | 7 (4.9) | 0 |
| No | 143 (94.7) | 137 (95.1) | 6 (85.7) |
| Prefer not to say | 1 (.7) | 0 | 1 (14.3) |
| **Anxiety Symptoms[a] (GAD-7)[b]** | (N=148) | n=141 | n=7 |
| None | 0 | 0 | 0 |
| Mild | 80 (54.1) | 76 (53.9) | 4 (57.1) |
| Moderate | 43 (29.1) | 42 (29.8) | 1 (14.3) |
| Severe | 25 (16.9) | 23 (16.3) | 2 (28.6) |

RB-TPP: Residence-Based Testing Participation Pilot; [a]PIP: Participated in RB-TPP; [b]Non-P: Did not participate in RB-TPP; [a] none (0-4), mild (5-9), moderate (10-14), and severe (15 and above). [b]Generalised Anxiety Disorders Scale – 7 item
